# Is hydroxychloroquine effective in treating primary Sjogren’s syndrome: a systematic review and meta-analysis

**DOI:** 10.1186/s12891-017-1543-z

**Published:** 2017-05-12

**Authors:** Shi-Qin Wang, Li-Wei Zhang, Pan Wei, Hong Hua

**Affiliations:** Department of Oral Medicine, Peking University School and Hospital of Stomatology & National Engineering Laboratory for Digital and Material Technology of Stomatology, 22 South Zhongguancun Avenue, Haidian District, Beijing, 100081 China

**Keywords:** Sjogren’s syndrome, Hydroxychloroquine, Systematic review, Meta-analysis

## Abstract

**Background:**

To systematically review and assess the efficacy and safety of hydroxychloroquine (HCQ) for treating primary Sjogren’s syndrome (pSS).

**Methods:**

Five electronic databases (Pubmed, EMBASE, Web of science, Ovid, Cochrane Library) were searched for randomized controlled trials and retrospective or prospective studies published in English that reported the effect of HCQ on pSS. The subjective symptoms (﻿sicca symptoms, fatigue and pain) and the objective indexes (erythrocyte sedimentation rate and Schirmer test) were assessed as main outcome measures. A meta-analysis and descriptive study on the efficacy and safety of HCQ were conducted. The estimate of the effect of HCQ treatment was expressed as a proportion together with 95% confidence interval, and plotted on a forest plot.

**Results:**

Four trials with totals of 215 SS patients, including two randomized controlled trials, one double blind crossover trial and one retrospective open-label study, were analyzed in this review. For dry mouth and dry eyes, the effectiveness of HCQ treatment was essentially the same as placebo treatment. For fatigue, the effectiveness of HCQ was lower than placebo. The efficacy of HCQ in treating pain associated with pSS was superior to that of the placebo. There was no significant difference between HCQ-treated groups and controls in terms of Schirmer test results, but HCQ could reduce the erythrocyte sedimentation rate compare with placebo. A descriptive safety assessment showed that gastrointestinal adverse effects were the most common adverse effects associated with HCQ.

**Conclusions:**

This systematic review showed that there is no significant difference between HCQ and placebo in the treatment of dry mouth and dry eye in pSS. Well-designed, randomized, controlled trials are needed to provide higher-quality evidence to confirm our findings, and future studies should focus on some other ﻿i﻿n﻿dex ﻿or﻿ extraglandular measures, such as cutaneous manifestations, to further explore the therapeutic effect of HCQ in pSS.

**Electronic supplementary material:**

The online version of this article (doi:10.1186/s12891-017-1543-z) contains supplementary material, which is available to authorized users.

## Background

Sjögren’s syndrome (SS) is a complex chronic autoimmune disease characterized by a wide spectrum of clinical manifestations. Lymphocytic infiltration and destruction of exocrine glands (mainly the salivary and lacrimal glands) is the histological hallmark of SS, leading to reduced lacrimal and salivary flow [1–3]. Dry eyes and dry mouth together with fatigue are among the most common complaints [4]. Extraglandular manifestations affect various organs including the skin, heart, lungs, kidney, gastrointestinal and endocrine system, as well as the central and peripheral nervous system [5–7]. SS with the presence of disorders mentioned above may occur alone without additional connective tissue diseases, as the primary SS (pSS), or may be associated to other autoimmune diseases, including systemic lupus erythematosus (SLE), rheumatoid arthritis (RA), and scleroderma, as secondary Sjogren’s syndrome (sSS) [8]. SS has an estimated prevalence of 0.5–4.8% [8–10], affecting approximately 1.5 to 4 million people in the USA based on a total population of 300 million [11]. The current epidemiological data indicate that the annual incidence rate of pSS is 6.92 people per 100,000 (95% confidence interval [CI]: 4.98 to 8.86). The prevalence rate is 60.82 people per 100,000 (95% CI: 43.69 to 77.94) [12]. According to previous report, the standardized mortality ratio ranges from 1.02 to 4.66, implying the impossible impact of pSS on patients' survival [13]. In most research, the increased mortality in pSS is mainly attributed to lymphoma [13]. The pooled risk ratio was 13.76 (95% CI 8.53 to 18.99) for non-Hodgkin lymphoma in pSS patients compared with the general populations [14].

The etiology and pathogenesis of this disease have not yet been elucidated, and it has been suggested that viral, hormonal, genetic, environmental, and neurophysiological factors might contribute to the initiation and progression of the disease [15–17]. Overexpression of proinflammatory cytokines, such as interleukin-1, and interleukin-17, and lower expression of anti-inflammatory cytokine, such as interleukin-4 is an important manifestation of immune disorder in SS [18, 19]. Recent advances in the understanding of its pathogenesis have uncovered some pathways that have potential as therapeutic targets. Activated B lymphocytes are a hallmark of the disease, which is also characterized by the presence of rheumatoid factor, hypergammaglobulinemia, and autoantibody to Ro/Sjogren’s-syndrome-related antigen A and La/Sjogren’s-syndrome-related antigen B [20]. In contrast, B cell depletion could result in normalization of the elevated levels of circulating follicular Th cells, which might be associated with lower systemic disease activity over time [21].

Among all chronic autoimmune rheumatic disorders, SS remains one of the most difficult to manage. At present, no curative agent exists [22]. Therapeutic goals remain symptom palliation, systemic damage prevention and life quality improvement. Suppression of an excessive abnormal immune response is key to critical patient care. The treatment method focuses on the disease process [23, 24]. When severe visceral damage is present, glucocorticoids or immunosuppressive therapy can be applied [23, 25]. Commonly applied immunosuppressive agents include tumor necrosis factor inhibitors, rituximab, hydroxychloroquine (HCQ), methotrexate and cyclophosphamide [22, 26].

HCQ is one of the first drugs applied in the treatment of rheumatism and plays an important role in alleviating fever. It controls arthritis and eliminates rash through complex mechanisms, such as anti-inflammation, immunosuppression, and immunomodulation [27, 28]. The efficacy of HCQ in the treatment of SLE [29] and RA [30] is already widely recognized, but its efficacy in the treatment of pSS is in dispute. Some studies have shown that it is effective in the treatment of pSS [31–33]. It is commonly used to treat fatigue, arthralgia, and myalgia [22, 34–36]. However, there are also studies that have come to the conclusion that it is not an effective treatment for pSS [37, 38]. Two recently published randomized controlled trials (RCTs) suggest that HCQ has no significant effect as compared with placebo [39, 40]. In the latest Sjogren’s Syndrome Foundation Clinical Practice Guidelines, HCQ is recommended as a first-line therapy for inflammatory musculoskeletal pain associated with pSS, and a moderate medication strength is recommended [41]. In terms of the recommended treatment for fatigue in pSS patients, HCQ may be considered for selective treatment of this symptom, and it is recommended to be administered at a weak strength. Currently, a systematic evaluation of the efficacy of HCQ in the treatment of pSS has not been conducted. This study is intended to provide a systematic evaluation of the efficacy and safety of HCQ in the treatment of pSS.

## Methods

This review was performed following the preferred reporting items for systematic reviews and meta-analyses (PRISMA) guidelines [42].

### Inclusion criteria

RCTs, retrospective studies, and prospective studies that used HCQ for pSS treatment were included in this review. There were no restrictions on patient age, sex, or race. The outcome measures included assessment for the remission of subjective symptoms such as dry mouth, dry eyes, pain, fatigue and the objective indexes, which contained erythrocyte sedimentation rate (ESR), Schirmer test and salivary flow rate. The diagnosis of SS was based on clinical assessment and laboratory examination, with or without histological evaluation.

### Exclusion criteria

For subjective symptoms, including sicca manifestations, pain and fatigue, dichotomous variables were used to evaluate the efficacy of HCQ treatment. If effective rate of HCQ treatment for subjective symptoms was not provided or not available according to original data, the research would be excluded. The continuous variables were used to assess the efficacy of HCQ for objective indices, control group was essential to conduct meta-analysis. Research regarding to objective indices without control group would be excluded from the analysis. Studies with a sample size of less than five patients were not included.

### Outcome evaluation

Outcome evaluation was based on primary and secondary outcomes. Primary outcomes were defined as the remission of the subjective symptoms of pSS, such as dry mouth, dry eyes, pain and fatigue. The effective rate would be calculated. Secondary outcomes were defined as the difference value before and after treatment of objective indexes, including ESR, Schirmer test and salivary flow rate. The adverse effects of interventions were also assessed.

### Database search strategies

All clinical trials in English that reported the effect on pSS of treatment with HCQ were selected in Pubmed, Excerpta Medica dataBASE (EMBASE), Web of science, Ovid and Cochrane Library. These databases were searched up to September 2016. The following combined terms were searched: (“Hydroxychloroquine” OR “Oxychlorochin” OR “Oxychloroquine” OR “Hydroxychlorochin” OR “Plaquenil”) AND (“Sjogren’s Syndrome” OR “Sjogrens Syndrome” OR “Sjogren Syndrome” OR “Sicca Syndrome”). Manual searches were also conducted as a supplement. The searches were conducted by two independent investigators (Shi-Qin Wang and Li-Wei Zhang).

### Data extraction and quality assessment

The two authors (Shi-Qin Wang and Li-Wei Zhang) were independently responsible for scanning titles and abstracts, selecting studies, reading full reports, extracting data, and assessing the quality of studies; these steps were performed in duplicate by each of these authors. A third reviewer (Hong Hua or Pan Wei) was invited to make an assessment if the two review authors could not reach a consensus. All the relevant data from each study including author, year of publication, characteristics of patients, detailed interventions, outcomes, and adverse effects were extracted and summarized in table format.

The quality of the studies was assessed using the Downs and Black quality assessment tool that contains a list of 27 criteria for evaluation of the reporting, external validity, internal validity-bias, confounding (selection bias), and the power of assessed studies [43]. The level of evidence represented by each study was categorized based on the Oxford Centre for Evidence-Based Medicine Levels of Evidence (OCEBM. http://www.cebm.net/index.aspx?o=5653). The OCEBM classifies the evidence levels of the research into five grades, with levels ranging from level 1 to level 5.

### Statistical analysis

The efficacy of HCQ was evaluated using StatsDirect 2.8.0 software (Stats-Direct Ltd, Altrincham, UK, 2013). The estimate of the effect of an intervention was expressed as a proportion together with a 95% confidence interval (CI), and plotted on a forest plot. The weighted mean difference was used for meta-analysis of continuous data. Cochran Q and I^2^ tests were performed to evaluate the heterogeneity of the studies. The random effect model was used when the test highlighted differences between studies, and the fixed-effect model was used when no significant differences were found [44]. When *P* < 0.05 and I^2^ > 0.25, the effect size heterogeneity was considered statistically significant [45].

## Results

### Identification of studies

**Fig. 1:**
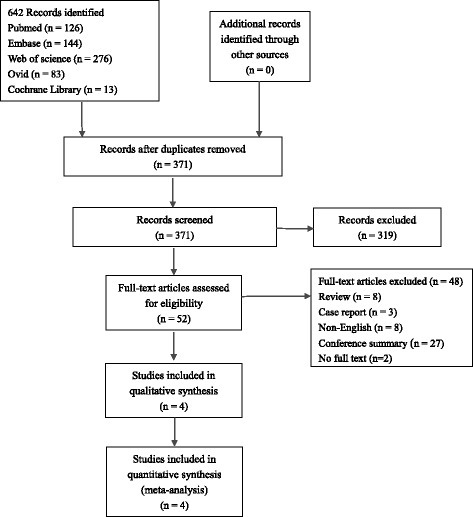
Flowchart showing the selection of articles

A total of 642 articles were obtained from five databases, 271 duplicates and 319 articles that did not meet the inclusion criteria were excluded. The remaining papers were analyzed, and from these, eight reviews, 27 conference summaries, three case reports, eight articles not in English and two articles with no pertinent full text were excluded. Finally, four studies [31, 37, 39, 40] were included in this review (Fig. 1).

### Characteristics of the studies

The characteristics of the four studies are summarized in Additional file 1. A total of 215 SS patients were included in this review. One [31] of the four studies did not use a control group. The sample size ranged from 19 to 120 patients in each study. The treatment duration ranged from 12 weeks to 2 years.

### Quality of studies

The median methodological quality score for all four studies, based on the Downs and Black criteria, was 28/32 (range 21 to 31). None of the included studies achieved a full score. In terms of the OCEBM Levels of Evidence, three studies [37, 39, 40] belonged to Level 2 and one study [31] belonged to Level 4 (Table 1).

**Table 1:** Quality assessment of included studies

Author	Year	Region	Study design	Downs and Black quality score						OCEBM levels of evidence
				Reporting (11)	External validity (3)	Internal validity - bias (7)	Internal validity - confounding (6)	Power (5)	Total (32)	
Yoon C.H. et al.	2016	Korea	RCT	9	3	6	6	5	29	Level 2
Gottenberg J.E. et al.	2014	France	RCT	10	3	7	6	5	31	Level 2
Fox R.I. et al.	1996	USA	Retrospective	10	0	3	3	5	21	Level 4
Kruize A.A. et al.	1993	Netherlands	Cross-over	10	2	7	5	3	27	Level 2

Abbreviations: *OCEBM* Oxford Centre for Evidence-Based Medicine, *RCT* randomized controlled trial

### Effects of HCQ

**Fig. 2:**
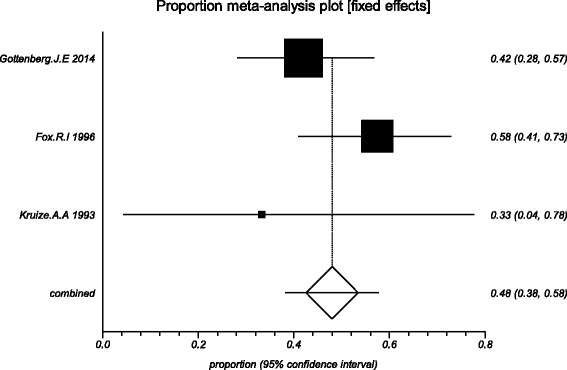
The efficacy of hydroxychloroquine treatment for dry mouth in primary Sjogren’s syndrome

**Fig. 3:**
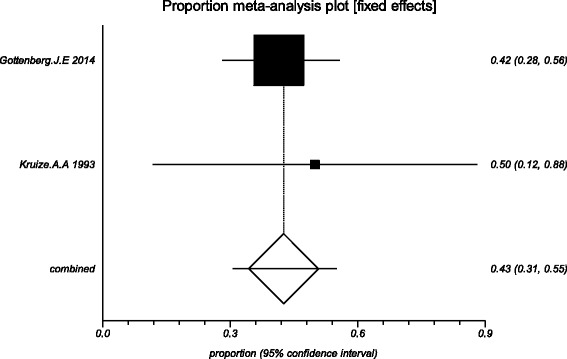
The efficacy of placebo treatment for dry mouth in primary Sjogren’s syndrome

**Fig. 4:**
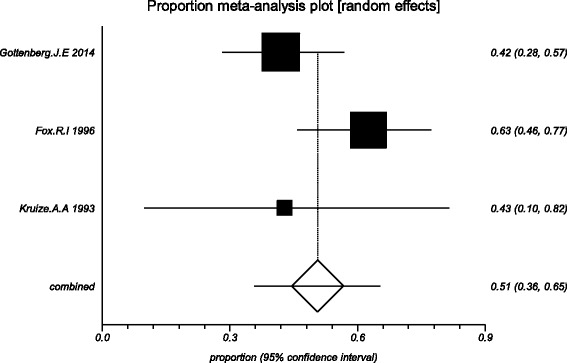
The efficacy of HQ treatment for dry eyes in primary Sjogren’s syndrome

**Fig. 5:**
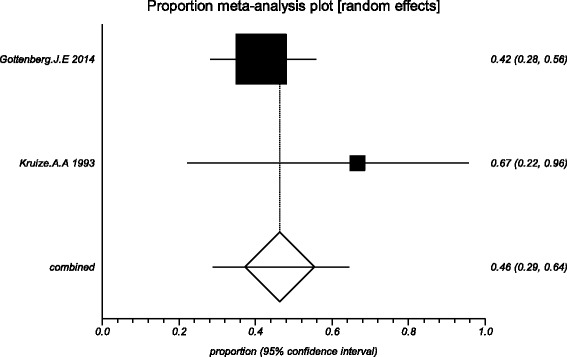
The efficacy of placebo treatment for dry eyes in primary Sjogren’s syndrome

**Fig. 6:**
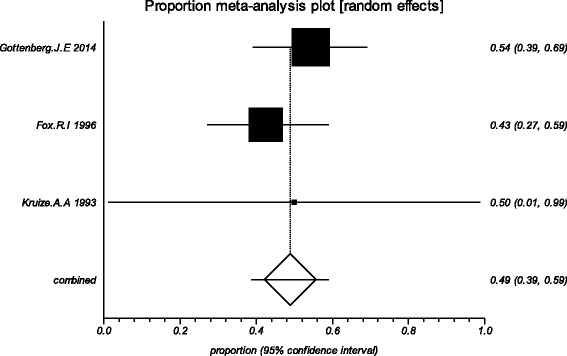
The efficacy of hydroxychloroquine treatment for pain in primary Sjogren’s syndrome

**Fig. 7:**
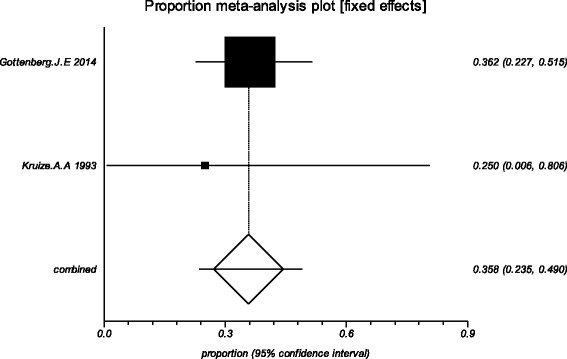
The efficacy of placebo treatment for pain in primary Sjogren’s syndrome

**Fig. 8:**
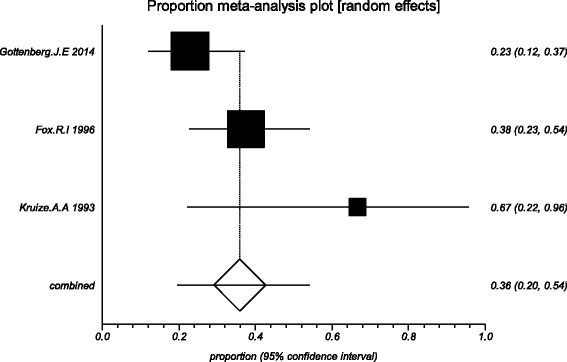
The efficacy of hydroxychloroquine treatment for fatigue in primary Sjogren’s syndrome

**Fig. 9:**
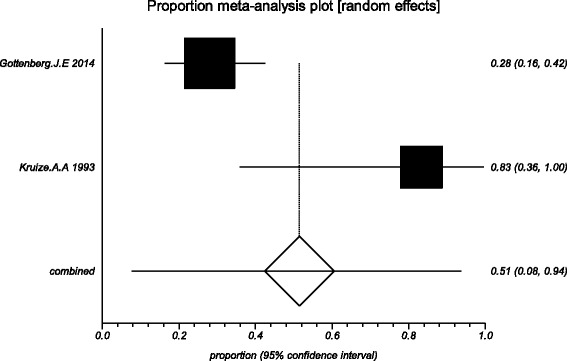
The efficacy of placebo treatment for fatigue in primary Sjogren’s syndrome

**Fig. 10:**
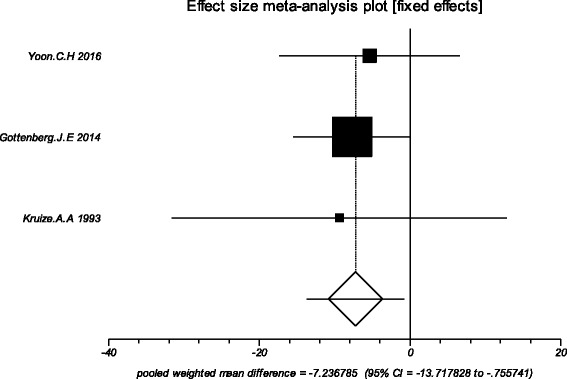
The pooled weighted mean difference of hydroxychloroquine versus placebo in erythrocyte sedimentation rate

**Fig. 11:**
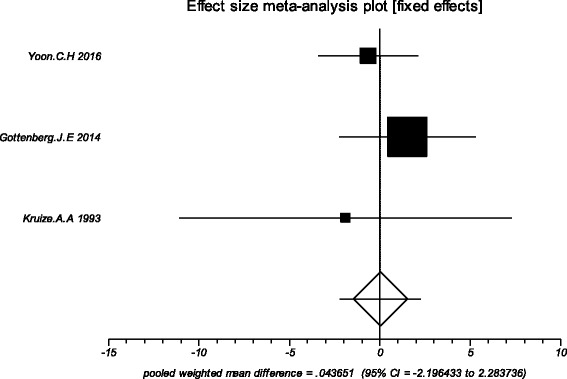
The pooled weighted mean difference of hydroxychloroquine versus placebo in the Schirmer test

The data from the included studies for meta-analysis are summarized in Table 2 and Table 3, and the results of the meta-analysis are shown in Tables 4 and 5. The pooled reduced proportions of subjective symptoms including dry mouth, dry eyes, pain and fatigue were analyzed (Table 4). For dry mouth, the efficacy of HCQ treatment (pooled proportion = 47.9%; 95% CI = 38.2–57.8%) was slightly higher than placebo treatment (pooled proportion = 42.6%; 95% CI = 30.6–55.1%), as depicted in Figs. 2 and 3. For dry eyes, the efficacy of HCQ treatment (pooled proportion = 50.6%; 95% CI = 40.8–60.3%) was higher than placebo (pooled proportion = 46.4%; 95% CI = 28.8–64.5%), as shown in Table 4, and Figs. 4 and 5. The results show that the efficacy of HCQ treatment (pooled proportion = 48.9%; 95% CI = 38.7–59.1%) for SS pain was higher than placebo treatment (pooled proportion = 35.8%; 95% CI = 23.5–49.0%; Table 4 and Figs. 6 and 7). For fatigue, the efficacy of HCQ (pooled proportion = 35.9%; 95% CI = 19.5–54.2%) was lower than that of the placebo (pooled proportion = 51.4%; 95% CI = 7.7–93.8%; Table 4 and Figs. 8 and 9). For the objective indexes, as salivary flow rate was only measured in one study which could not be used for meta-analysis, so only the pooled weighted mean difference of the ESR and Schirmer test were analyzed (Table 5), and it was found that HCQ treatment could reduce the ESR of SS patients (Z = −2.19, *P* < 0.05; Fig. 10), however, there was no statistically significant difference in Schirmer test (Z = 0.04, *P* = 0.97; Fig. 11).

**Table 2:** Data of subjective symptoms and adverse effects or serious adverse events in the included studies

Author	Year	Region	Total No. and No. of events	Hydroxychloroquine	Placebo
Gottenberg J.E. et al.	2014	France	Total patients	56	64
			No. of dry mouth	50	53
			No. of improved (%)	21 (42.0)	22 (41.5)
			No. of dry eyes	50	53
			No. of improved (%)	21 (42.0)	22 (41.5)
			No. of pain	46	47
			No. of improved (%)	25 (54.3)	17 (36.2)
			No. of fatigue	48	50
			No. of improved (%)	11 (22.9)	14 (28.0)
			No. of adverse effects	-	-
			No. of serious adverse events	2	3
Fox R.I. et al.	1996	USA	Total patients	50	-
			No. of dry mouth	40	-
			No. of improved (%)	23 (57.5)	-
			No. of dry eyes	40	-
			No. of improved (%)	25 (62.5)	-
			No. of pain	40	-
			No. of improved (%)	17 (42.5)	-
			No. of fatigue	40	-
			No. of improved (%)	15 (37.5)	-
			No. of adverse effects	8	-
			No. of serious adverse events	-	-
Kruize A.A. et al.	1993	Netherlands	Total patients	10	9
			No. of dry mouth	6	6
			No. of improved (%)	2 (33.3)	3 (50.0)
			No. of dry eyes	7	6
			No. of improved (%)	3 (42.9)	4 (66.7)
			No. of pain	2	4
			No. of improved (%)	1 (50.0)	1 (25.0)
			No. of fatigue	6	6
			No. of improved (%)	4 (66.7)	5 (83.3)
			No. of adverse effects	1	0
			No. of serious adverse events	-	-

**Table 3:** Data of erythrocyte sedimentation rate and Schirmer test results in the included studies

Author	Year	Region	Outcomes	Hydroxychloroquine			Placebo		
				N	Mean difference	sd	N	Mean difference	sd
Yoon C.H et al.	2016	Korea	ESR	11	-3.64	19.30	15	1.73	11.75
			Schirmer test	11	-1.45	4.31	15	-0.80	2.89
Gottenberg J.E et al.	2014	France	ESR	44	-4.0	22.3	48	3.7	15.5
			Schirmer test	37	2.14	8.83	36	0.62	7.50
Kruize A.A et al.	1993	Netherlands	ESR	8	-10.3	17.1	6	-0.9	25.5
			Schirmer test	8	-1.6	9.4	6	0.3	7.6

Abbreviations: *ESR* erythrocyte sedimentation rate

**Table 4:** Results of the meta-analysis concerning subjective symptoms

	Hydroxychloroquine	Placebo
Total, dry mouth		
No. of studies	3	2
Total patients observed	96	59
No. of improved	48	25
Pooled proportion, % (95% CI)	47.9 (38.2 to 57.8)	42.6 (30.6 to 55.1)
I^2^ Inconsistency	22.6	-
Total, dry eyes		
No. of studies	3	2
Total patients observed	97	59
No. of improved	49	26
Pooled proportion, % (95% CI)	50.6 (40.8 to 60.3)	46.4 (28.8 to 64.5)
I^2^ Inconsistency	48.4	-
Total, pain		
No. of studies	3	2
Total patients observed	88	51
No. of improved	43	18
Pooled proportion, % (95% CI)	48.9 (38.7 to 59.1)	35.8 (23.5 to 49.0)
I^2^ Inconsistency	0	-
Total, fatigue		
No. of studies	3	2
Total patients observed	94	56
No. of improved	30	19
Pooled proportion, % (95% CI)	35.9 (19.5 to 54.2)	51.4 (7.7 to 93.8)
I^2^ Inconsistency	63.4	-

**Table 5:** Results of the meta-analysis: erythrocyte sedimentation rate and Schirmer test

Test	Control	Study number	Patient number			Pooled effect size wmd (95% CI)	*P*-value	I^2^ (%)
			HCQ group	Control group	Total			
ESR	Placebo	3	63	69	132	−7.24 (−13.72 to -0.76)	0.03	0.0
Schirmer test	Placebo	3	56	57	113	0.04 (−2.20 to 2.28)	0.97	0.0

Abbreviations: *ESR* erythrocyte sedimentation rate, *HCQ* Hydroxychloroquine, *CI* confidence interval; wmd, weighted mean difference

### Safety assessment

Among the four articles included in this review, three [31, 37, 39] reported adverse effects or serious adverse events associated with HCQ treatment for pSS. Among the 116 patients in the HCQ group, there were nine adverse effects and two serious adverse events. One study [31] did not show the details of the adverse effects and only the number of adverse effects was reported. In the current systematic review, the most common adverse effects were gastrointestinal side effects, which occurred in five among the nine patients who had side effects after HCQ treatment. In the study by Kruize et al. [37], one patient demonstrated liver damage after receiving HCQ. In the study by Gottenberg et al. [39], there were two (urinary lithiasis, breast cancer) serious adverse events in the HCQ group and three (surgery for meningioma, lipothymia, Epstein-Barr virus (EBV) and cytomegalovirus (CMV) pneumonia) in the placebo group (Table 2).

## Discussion

In this meta-analysis, four trials with totals of 215 SS patients were analyzed. The efficacy of HCQ in SS was not superior or even inferior to placebo regarding to the subjective symptoms, including dry mouth, dry eyes and fatigue. The effectiveness of HCQ in treating pain associated with pSS was higher than that of the placebo. And HCQ treatment could also reduce the ESR of pSS patients,however, when considering the treatment result of Schirmer test, no significant difference between HCQ and control groups was found. Gastrointestinal adverse effects were the most common adverse effects among patients treated with HCQ.

HCQ was synthesized by Surrey and Hammer in 1950. It can control arthritis and eliminate rash through its anti-inflammatory, immunosuppressive, and immunomodulatory functions. For approximately 70 years, HCQ has been employed for the treatment of SLE and RA [28], and the efficacy of HCQ in the treatment of SLE [29] and RA [30] is widely recognized. The earliest study on the treatment of SS with HCQ was published in 1963 [46], and although there was a long history of research on the subject, the majority of studies were not conducted until the 1990s [47–49]. HCQ is widely used in clinical practice to treat SS [50], but its efficacy for this application is still controversial. Some studies [31, 33, 51, 52] indicate that HCQ is effective in the treatment of SS. In 1988, Fox et al. [51] observed that the total immunoglobulin G of SS patients significantly decreased in a HCQ treatment group. Another study conducted by Fox et al. in 1996 [31] reported that a sustained improvement of local symptoms (painful eyes, painful mouth) and an improvement in systemic manifestations (arthralgias and myalgias) in pSS patients were found after treatment with HCQ. In an open label study conducted by Tishler et al. indicated that HCQ could significantly reduce some salivary inflammatory markers in SS patients [32]. In 2011, Yavuz et al. [52] demonstrated that HCQ may alleviate the signs and symptoms of dry eyes in pSS and decrease tear fluid B-cell activator factor (BAFF) levels. In 2013, Mumcu et al. [33] showed that salivary and serum BAFF levels were lowered in patients with pSS when treated with HCQ, and also that decreased disease activity and increased salivary flow could be achieved using HCQ treatment in pSS patients.

However, other studies [37–40] have demonstrated that HCQ treatment in SS is ineffective. In particular, two recent RCT studies have found that HCQ had a very limited effect on SS. In 2014, Gottenberg et al. [39] found that among patients with pSS, the use of HCQ did not improve symptoms compared with placebo during 24 weeks of treatment. In 2016, Chang et al. [40] showed that HCQ at 300 mg daily for 12 weeks had no apparent clinical benefit for dry eyes and systemic inflammation in pSS. The Sjogren’s Syndrome Foundation Clinical Practice Guidelines currently recommend the use of HCQ to treat some of the symptoms of SS [41]. However, there is currently no evidence-based medical proof to support this approach. This is the first study to perform a systematic evaluation of the safety and efficacy of HCQ in the treatment of SS.

Of the four studies included in this research, three [31, 37, 39] adopted different scales to evaluate the efficacy of HCQ in treating the subjective symptoms of pSS. As can be seen from the combined results of these three studies, the efficacy of HCQ for dry mouth was slightly higher than placebo. However, only one [39] of these was a high-quality RCT study, which showed no statistically significant difference between HCQ and placebo in relieving dryness symptoms. This RCT study [39] also measured unstimulated salivary flow rate. The results show that there was no significant difference in the amount of saliva secreted between patients treated with HCQ and placebo, which is consistent with the result of another case series study [38]. Similarly, in terms of dry eyes, the combined results of the three studies [31, 37, 39] show that HCQ produced a slightly higher response rate than placebo, but the only RCT study showed no statistically significant difference. In addition, one RCT study used ocular surface disease index (OSDI) to evaluate the symptoms of dry eye after HCQ treatment in patients with SS, and the results showed there were no statistically significant difference between HCQ and placebo in relieving dryness symptoms [40]. Furthermore, three [37, 39, 40] of the four studies reported Schirmer test results. Combining the results of these three studies [37, 39, 40], the Schirmer test results revealed no significant difference between the impact of HCQ and placebo. Two [39, 40] of these three studies were high-quality RCT studies, and the results of the current meta-analysis show no heterogeneity. Therefore, HCQ has extremely limited efficacy in the treatment of dry mouth and dry eyes based on the current published articles.

Three [31, 37, 39] of the four studies reported on the efficacy of HCQ in treating pain and fatigue. The combined results of the studies show that HCQ was more effective in relieving pain associated with pSS than placebo; however, HCQ was somewhat less effective than placebo in relieving fatigue in pSS patients. The only RCT study [39] showed that there was no statistically significant difference between HCQ and placebo in alleviating fatigue and pain. Furthermore, three [37, 39, 40] of the four studies included in the current analysis reported the results of ESR tests. The combining results of these three studies showed that when compared with placebo group, HCQ treatment could effectively reduce ESR in patients with pSS. Therefore, it was concluded that HCQ had no effect upon pain, fatigue, but HCQ could reduce the ESR of pSS patients.

With regard to the safety of HCQ in the treatment of SS, three [31, 37, 39] of the four studies recorded side effects or adverse events after treatment. Among the 116 patients in the HCQ group, there were nine adverse effects and two adverse events, while among the 73 patients in the placebo group, there were three adverse events but no adverse effects. Of the nine adverse effects in the HCQ group, eight occurred in patients who participated in the same study [31], among which, five patients suffered gastrointestinal issues, and three patients suffered a rash attributed to the drug, and this study did not set placebo group. In the study by Kruize et al. [22], one patient demonstrated liver damage after receiving HCQ. In the study by Gottenberg et al. [24], there were two (urinary lithiasis, breast cancer) serious adverse events in the HCQ group (56 patients) and three (surgery for meningioma, lipothymia, EBV and CMV pneumonia) in the placebo group (64 patients). The occurrence of adverse events in the HCQ group (3.6%) was not significantly different to that in the placebo group (4.7%). Therefore, these results suggest that HCQ is safe for use in the treatment of pSS.

Although the present study provided an objective and comprehensive evaluation of the safety and efficacy of HCQ in the treatment of pSS, there are some limitations that need to be addressed. Firstly, all the included literature was in English, and therefore literature in other languages may have been overlooked. Secondly, relatively few studies were included. A total of four studies were included, of which only two were RCT studies, leading to a lower level of evidence. Thirdly, there were slight differences in the dosage and course of treatment among the four studies, which may impact prognosis to a certain extent. Fourth, subjective symptoms were evaluated and each study used a different scale for evaluation, which may also influence the results. Lastly, considering the results are largely determined by one ﻿or two researchs due to its sample size, the underlying bias should not be ignored. Based on the above limitations, a full analysis of the objective effects of HCQ in the treatment of pSS should be performed and the clinical role of HCQ should not be expanded. Future high-quality RCTs with large populations are essential to confirm the efficacy of HCQ for treating pSS.

## Conclusion

In summary, the efficacy of HCQ to alleviate the sicca manifestations of pSS, based on current published studies, is extremely limited. Considering the wide use of HCQ in treating pSS, well-designed, randomized, controlled trials are needed to provide higher-quality evidence to confirm our findings, and future studies should focus on other outcome measures, such as cutaneous manifestations et al, to objectively and comprehensively explore the role of HCQ in the treatment of pSS.

## Additional file

Additional file 1: An additional file shows the characteristics of the included studies. (DOC 33 kb)

## Abbreviations

BAFF: B-cell activator factor
CI: Confidence interval
CMV: Cytomegalovirus
EBV: Epstein-Barr virus
EMBASE: Excerpta Medica Database
ESR: Erythrocyte sedimentation rate
HCQ: Hydroxychloroquine
OCEBM: Oxford centre for evidence-based medicine
pSS: Primary Sjogren’s syndrome
RA: Rheumatoid arthritis
RCTs: Randomized controlled trials
SLE: Systemic lupus erythematosus
SS: Sjogren’s syndrome
sSS: Secondary Sjogren’s syndrome
wmd: Weighted mean difference
