# Supplementary material for: Is hydroxychloroquine effective in treating primary Sjogren’s syndrome: a systematic review and meta-analysis
Source: BMC Musculoskelet Disord. 2017 May 12;18:186. doi: 10.1186/s12891-017-1543-z (PMC5427554; doi:10.1186/s12891-017-1543-z)
Supplement: Additional file 1: — An additional file shows the characteristics of the included studies. (DOC 33 kb) [file 12891_2017_1543_MOESM1_ESM.doc]

| **Author** | **Year** | **Region** | **Diagnostic criteria** | **HCQ group** | | | | **Control group** | | | | **Dosage** | **Duration** | **Follow-up** |
| --- | --- | --- | --- | --- | --- | --- | --- | --- | --- | --- | --- | --- | --- | --- |
|  |  |  |  | **Age** | **Sex ratio** | **N** | **Medication** | **Age** | **Sex ratio** | **N** | **Medication** |  |  |  |
|  |  |  |  | **(mean)** | **(F:M)** |  |  | **(mean)** | **(F:M)** |  |  |  |  |  |
| Yoon C.H  et al. | 2016 | Korea | AECG (2002) | 59.4 | 100:0 | 11 | HQ | 55.0 | 100:0 | 15 | placebo | 300 mg/d | 12 w | 16 w |
| Gottenberg J.E  et al. | 2014 | France | AECG (2002) | 56.3 | 89.3:10.7 | 56 | HQ | 55.6 | 93.8:6.2 | 64 | placebo | 400 mg/d | 24 w | 48 w |
| Fox R.I  et al. | 1996 | USA | San Diego (1994) | 49.7 | 100:0 | 50 | HQ | – | – | – | – | 6–7 mg/kg/d | 2 y | 2 y |
| Kruize A.A  et al. | 1993 | Netherlands | Daniels and Talal (1987) | 52.8 | 100:0 | 10 | HQ | 51.0 | 100:0 | 9 | placebo | 400 mg/d | 1 y | 2 y |

**Additional file 1.** Characteristics of the included studies

**Abbreviations: HCQ,** **Hydroxychloroquine; AECG, American-European Consensus Group; d, days; w, weeks; y, years; F, female; M, male.**
